# Insular dysfunction of interoception in major depressive disorder: from the perspective of neuroimaging

**DOI:** 10.3389/fpsyt.2023.1273439

**Published:** 2023-09-28

**Authors:** Lan Hu, Hui He, Neil Roberts, Jiajia Chen, Guojian Yan, Li Pu, Xufeng Song, Cheng Luo

**Affiliations:** ^1^The Clinical Hospital of Chengdu Brain Science Institute, MOE Key Lab for Neuroinformation, University of Electronic Science and Technology of China, Chengdu, China; ^2^The Fourth People’s Hospital of Chengdu, Chengdu, China; ^3^Centre for Reproductive Health (CRH), School of Clinical Sciences, University of Edinburgh, Edinburgh, United Kingdom

**Keywords:** major depressive disorder, insula, neuroimaging, interoception, functional magnetic resonance imaging

## Abstract

Interoception plays a crucial role in maintaining bodily homeostasis and promoting survival, and is considered the basis of human emotion, cognition, and self-formation. A malfunction of interoception is increasingly suggested to be a fundamental component of different mental health conditions, and depressive disorders have been especially closely associated. Interoceptive signaling and processing depends on a system called the “interoceptive pathway,” with the insula, located in the deep part of the lateral fissure, being the most important brain structure in this pathway. Neuroimaging studies have revealed alterations in the structure and function of the insula in a large number of individuals with depression, yet the precise relationship between these alterations and interoceptive dysfunction remains unclear. The goal of this review is to examine the evidence that exists for dysfunction of interoception in people with Major Depressive Disorder (MDD), and to determine the associated specific alterations in the structure and function of the insula revealed by neuroimaging. Overall, three aspects of the potential relationship between interoceptive dysfunction and alterations in insular function in people with depression have been assessed, namely clinical symptoms, quantitative measures of interoceptive function and ability, and interoceptive modulation. To conclude, several specific limitations of the published studies and important lines of enquiry for future research are offered.

## 1. What is interoception and what is its association with major depressive disorder (MDD)?

The concept of interoception was first put forward over 100 years ago by Sherrington who proposed that certain sensory receptors are preferentially excited by so-called interoceptive stimuli that originate, for example, within the viscera of the body (1). In 2002, Craig redefined interoception as pertaining to the physiological condition of the entire body (2). The signals not just came from viscera, but also include temperature, pain, itch, tickle, sensual touch, muscular sensations, vasomotor flush, hunger, thirst, and other sensations related to the body’s state. Based on the anatomy of the ascending spinal laminar 1 spinothalamic tract, Craig proposed a new and fundamental framework for connecting interoception with subject emotion, motivation and consciousness. In particular, according to the framework proposed by Chen (3), interoception concerns the sensing, interpretation, integration and regulation of all these signals. Additionally, Chen (3) has emphasized how the signal processing between the brain and the internal organs is bidirectional, and this expanded framework offers a more comprehensive perspective of the dynamic interoceptive processes, greatly advancing the emerging field of interoception.

The proposal that there is a link between interoception and emotions was also made over 100 years ago. In particular, according to the James–Lange theory emotional feelings are secondary phenomena produced by perceptions arising from somatic and visceral changes (4). Appreciation of the fundamental role played by perception of body-wide physiological changes in constituting emotional feelings, has had a profound effect on subsequent scientific research as reviewed by (5). For example, towards the end of the 20th century, Domasio proposed the so-called somatic marker hypothesis in which the key role of bodily responses (e.g., visceral events, gland secretion, skeletal muscle movement, etc.) in the formation of emotions and impacts on decision making is highlighted (6). Evidence has also been accumulating to suggest that there is an important link between somatic symptoms and emotion in clinical conditions (e.g., pain, fatigue and appetite dysregulation in MDD) (7). The feelings people perceive from their bodies inevitably affects their state of mood and overall sense of well-being (8).

MDD is a mental disorder that is manifested mainly by emotional symptoms such as low mood and decreased interest, and with high frequency of somatic symptoms. Clinical signs and symptoms, such as increased or decreased appetite, and chronic pain and fatigue, have been observed and interpreted as indicating that there is dysfunction of interoception in MDD. In order to allow objective study of the underlying relationships, in the last decade scientists have developed several quantitative measures for assessing the interoceptive functional abilities of an individual. Garfinkel and Critchley (9) proposed that there exist three distinct dimensions of interoception, namely, (i) objective interoceptive sensitivity which refers to one’s objective accuracy in detecting internal bodily sensations, (ii) subjective interoceptive sensibility which refers to one’s self-reported belief about one’s interoceptive accuracy, and (iii) interoceptive awareness which refers to metacognitive awareness of one’s interoceptive accuracy. Subsequently, the same authors demonstrated how these dimensions can be measured (10). Most studies have concerned the measurement of interoceptive sensitivity and cardiac-based measures of heartbeat perception are a commonly used task paradigm. For example, studies have been performed to investigate how accurately individuals can perform heartbeat tracking and heartbeat discrimination tasks. In a recent systematic review, it was reported that the level of accuracy of interoceptive measurement of heartbeat was associated with the severity of symptoms of depression in patients with MDD (11). In addition, research performed using a self reporting approach has also provided evidence of dysfunction in interoceptive sensibility and awareness in individuals with depression (12–14). For example, recent studies using the Multidimensional Assessment of Interoceptive Awareness (MAIA) questionnaire to record eight facets (i.e., Noticing, Not-Distracting, Not-Worrying, Attention Regulation, Emotional Awareness, Self-Regulation, Body Listening, Trusting) of interoception, found inverse associations between the severity of depression and scores in several of the MAIA sub-scales (i.e., Attention Regulation, Trusting, Not-Worrying), indicating multidimensional impairments in interoception in patients with MDD (12, 15). Eggart et al. (12) further found that all dimensions of interoceptive function improved by treatment with anti-depressive medication, highlighting the potential of measuring interoception to predict clinical outcome of treatment of depression. Some researchers have expressed concerns that the three dimensional construct of Garfinkel and Critchley (9) concerns only explicit interoception and is insufficient to describe all aspects of an individual’s interoceptive ability. In particular, subconscious perception of one’s internal states (i.e., implicit interoception) may also impact on the regulation of the bodily states (16). Whether explicit or implicit, abnormalities of the neural pathways of interoceptive processing are thought to be the foundation for interoceptive dysfunction.

## 2. The role of the insula in interoception

In 1955, Canadian Neurosurgeon Wilder Penfield applied electrical stimulation to different areas of the cerebral cortex in patients during surgery (17). He found that stimulation of insular cortex gave rise to a series of visceral sensations and movements, leading him to propose that the insula is the primary interoceptive cortex. Subsequent research has demonstrated that damage to the insula, such as that caused by stroke or tumors, can be associated with changes in internal states, including cardiac arrhythmia (18), altered blood pressure patterns (19), cardiomyopathy (20, 21), deficits in taste recognition and intensity (22), and temperature perception (23). Non-invasive neuroimaging research in humans in which the insula has been shown to be activated by numerous interoceptive modalities such as thermal pain, C-fibre touch and graded cooling has provided further evidence to support the role of the insula in monitoring bodily state (2).

Anatomically, the insula is connected with sub-cortical regions involved in interoceptive integration (e.g., Nucleus Tractus Solitarius (NST) otherwise known as the Solitary Tract, hypothalamus, and parabrachial nucleus) and serves as the cortical site for anatomical convergence of interoceptive inputs from spinal cord lamina 1 and NST (2, 16, 24). Specifically, the posterior insula receives primary interoceptive information directly from afferent projections to subcortical structures where peripheral information regarding bodily state, delivered through the humoral, lymphatic, or peripheral nervous systems, is integrated (3, 25). This primary interoceptive information is then sent rostrally to anterior insula for further integration with exteroceptive, emotional and motivational information. With regard to the specific roles of left and right insula there still remains some debate, with most studies showing evidence that interoceptive information is mainly processed by right insula (26–28). However, there is also evidence that interoceptive processing activates the insula bilaterally (29).

Tracer studies performed in both human and non-human primates have revealed a wide array of connections between the insula and the majority of other cortical regions, including frontal, temporal, parietal and occipital lobes as well as limbic regions (30). In addition, neuroimaging has provided evidence that the insula is a rich ‘hub’ of large-scale brain networks and serves as an interface between cognitive, homeostatic, salience and affective systems of the human brain (31), providing further evidence to support the influence of interoception on the shape of cognition, emotions and behavior (24, 25, 32). Given the central role of the insula in interoception, any deficits in the structure or function of the insula may lead to abnormal representations of bodily signals, and have a varying degree of impact on mental activity and potentially lead to mental disorders.

## 3. Insular dysfunction and interoception impairment in MDD

Reports of structural and functional impairments in the insula of individuals with MDD have been documented in various studies (33–37). However, it is not known whether these impairments of the insula may be a cause or a consequence of interoceptive dysfunction in people with MDD. In this section, we will discuss the potential role of insular changes in the context of interoceptive dysfunction in depressive disorder from three aspects, namely symptomatology, measurement, and modulation.

### 3.1. Symptomatology

People with MDD are frequently found to have diverse co-morbid chronic conditions such as irritable bowel syndrome and chronic fatigue syndrome which are known as functional diseases. A recent pilot study demonstrated interoceptive difficulties occur in patients with somatic symptom disorder (38). Non-specific somatic symptoms have also often been recognized as signs that a person may be depressed. Harshaw (39) reviewed the close association between somatic symptoms such as headache, fatigue, sleep alterations and appetite changes, and interoceptive dysfunction in people with depression, proposing that the somatic symptoms might be mediated by inflammatory cytokines, vagal tone, gut signals, etc. Abnormalities of the insula are recognized as a sign of potential interoceptive dysfunction, and interestingly, several neuroimaging studies have shown that a correlation exists between the severity of somatic symptoms and insular changes. For example, in one study of people with depression using resting-state functional magnetic resonance imaging (fMRI), it was revealed that reduced functional brain connectivity between left insula and left centromedial amygdala was positively correlated with severity of somatic symptoms (40), while in another functional connectivity between ventral anterior insula and right orbitofrontal cortex was negatively correlated with severity of somatic symptoms, but returned to normal following treatment with electroconvulsive therapy which ameliorated somatic symptoms (41). Overall, it is reasonable to infer that insular changes associated with somatic symptoms in people with depression might be an indication of interoceptive dysfunction.

Fatigue is the most common non-specific somatic symptom occurring in people with MDD and there is a high prevalence of residual impairment even after treatment with antidepressants. Fatigue has also been reported to be linked to structural or functional impairments of the insula, or altered insula connectivity, in diverse diseases, such as cancer (42) ankylosing spondylitis (43), multiple sclerosis (44, 45), Parkinson’s disease (46), and traumatic brain injury (47). In the case of depression, training in TaiChi over a period of 10 weeks, and which was predicted to enhance interoceptive function, produced improvements in fatigue and mood (48). The fact that enhancements of insular functional connectivity were also observed leads to the potential interpretation that fatigue in depression is mediated by interoceptive dysfunction and inflammation is one possible mechanism underlying fatigue in several conditions including depression (25, 49) and there are three facts that support this interpretation.

Firstly, a possible close association between inflammatory changes and interoceptive disturbances is consistent with the immune system being a diffuse sensory ‘organ’ encompassing the whole body (50). Neural, humoral and cellular interoceptive signaling pathways all play a critical role in communicating the state of the immune system to the brain to trigger appropriate responses in motivation, mood and cognition. Furthermore, activation of insula and thalamus, brain regions which belong to the interoceptive pathway, has been reported in several neuroimaging studies in which inflammation has been experimentally induced systemically in humans, such as by endotoxin administration (51), typhoid vaccination (52, 53), and lipopolysaccharide injection (54). Long-term training in TaiChi is claimed to enhance interoception function and in a recent meta-analysis was shown to effectively reduce levels of inflammatory factors, including tumor necrosis factor-α, interleukin-6 and C-reactive protein (55).

Secondly, inflammation may play an etiologic role in the pathogenesis of fatigue leading to depression. For example, increased levels of peripheral and neural inflammatory markers were found to be present in people with depression (56, 57), and prolonged interferon-alpha therapy, endotoxin administration, and a *Salmonella typhi* vaccine were observed to trigger symptoms of depression and/or fatigue in humans (51, 58, 59). Also it has been demonstrated that treatment of mice with tumor-induced IL-1β expression in the brain with minocycline produced a reduction in depression and fatigue behaviors (60).

Thirdly, there may be a neuroimmune basis underlying the association between inflammation-induced interoceptive dysfunction and fatigue and/or symptoms of depression. This suggestion is based on results from brain imaging studies of patients with fatigue and other symptoms of depression attributed to systemic inflammation induced by vaccine or endotoxin who show increased glucose metabolism of the insula (51) and augmented posterior–anterior effective connectivity within the insula (59). However, more direct evidence is needed if the specific neuroimmune mechanism is to be determined, such as measures of local metabolism and activity and intra- and inter-insula connectivity and network properties, and knowledge of the specific impairments in interoception that occur in people with depression.

Pain is another common clinical co-morbidity occurring in people with depression and which may be related to impairment in interoceptive function (61). The results of a meta-analysis show that compared to healthy controls people with depression had significantly reduced pain threshold and sensory tolerance to ischemic pain produced, for example, by an experimental tourniquet procedure (62). These findings are concordant with frequent reports of experiencing non-specific pain made by people with depression. Ischemic pain, together with non-specific pain from muscles and joints, and thermal pain, visceral pain, etc., are all evoked by nociceptors belonging to the interoceptive pathway and which is more severe and diffuse, and more vulnerable to negative affect, than other pain modalities (62, 63). Antidepressants are an effective clinical treatment for reducing interoceptive pain (64).

The insula has long been recognized as a core region involved in processing the perceptual, affective, and cognitive response to multiple pain modalities (65). The results of a recent study support an interplay of sad mood and inflammation on brain regions involving the insula during the expectation and experience of visceral pain induced by extraneous lipopolysaccharide (66). However, experimentally, typical or conventional interoceptive pain, usually referred to as visceral pain, is very difficult to induce. Thermal pain is therefore the pain modality that has been most commonly investigated in patients with psychiatric disorders. Although it is conventional to regard thermal pain as a kind of exteroceptive pain, Craig (2) redefined and broadened the concept of interoception based on the functional anatomy of the lamina I spinothalamocortical system, and classified thermal pain as interoceptive. Converging evidence from neuroimaging studies has shown that neural processing of thermal pain is altered in people with depression. For example, studies using an experimental thermal pain paradigm have repeatedly shown increased insular reactivity, specifically in the anterior part, when people with depression experience painful heat stimuli compared to healthy controls (67, 68). In addition, functional connectivity between dorsal insula and posterior thalamus was higher, and between dorsal insula and right inferior frontal gyrus was lower, when people with MDD experience painful heat stimuli compared to healthy controls, suggesting greater reactivity of the interoceptive pathway and less regulation by the frontal brain system in people with MDD (67). A close association between sensitivity to thermal pain and depressive mood has also been revealed in healthy subjects and in patients with other diseases, and heightened insular reactivity was found to be an important component mediating this association (69–71). One possible explanation for these findings is that increased concentration of the excitatory neurotransmitter glutamate and its precursor glutamine in brain regions involved in processing interoceptive stimuli, and which includes the insula, may cause increased pain sensitivity and symptoms of depression (72). Interestingly, a meta-analyses of neuroimaging studies of the processing of emotion revealed there to be a functional reorganization of the insula in people with MDD such that activation is topologically shifted towards the anterior dorsal part of the insula which is involved in pain processing in healthy individuals. This may explain why people with MDD experience pain in response to stimuli that are normally not painful and be more prone to pain-related emotion (65).

The meta-analyses of resting-state fMRI studies have shown there to be significantly increased amplitude of low-frequency fluctuations (ALFF) (73, 74) and significantly increased regional cerebral blood flow (rCBF) (73) in the insula in people with MDD compared to healthy controls. Altered baseline activity of insula in MDD may thus be a marker of interoceptive dysfunction. Moreover, a recent fMRI study showed that depressive symptoms of HIV patients were positively associated with the magnitude of blood oxygen level-dependent (BOLD) activation in bilateral insula while the patients were performing a heartbeat detection task during scanning, supporting the evidence that interoceptive-related insular activity may reflect a feature of depression (75). Further studies using additional experimental paradigms to study the role of interoceptive dysfunction in the development of various depressive symptoms, such as appetite loss, sleep disorders and other somatic dimensions, are warranted.

### 3.2. Measurement of interoception

The development of methods for measuring the interoceptive function and ability of an individual is important for investigating corresponding changes in disease states and determining their underlying biological mechanism. At the present time, objective or subjective measurement of interoception is almost entirely based on heartbeat counting or detection, and requires a person to count the number, or provide a description, of their heartbeats occurring in a specific time.

An alternative approach is to use neuroimaging, and since the insula is known to play an integral role in interoception and emotion, this brain structure is of special interest for study. In the resting state, close intrinsic functional connectivity of sub-regions of the insula within the salience network and with regions, such as orbitofrontal cortex and prefrontal cortex, has been found to be associated with higher cardiac-based interoceptive accuracy (76, 77). Furthermore, females exhibited higher performance on a scale measuring subjective interoceptive sensibility and were also found to possess larger insular volume bilaterally compared to males, and in addition exhibited a positive correlation between grey matter volume of left insula and interoceptive sensibility (78). These findings that the volume of insula may be a key determinant of interoceptive ability are supported by the results of an fMRI study in which interoceptive accuracy was found to be positively related to insula activation when subjects were required to pay attention to their heartbeat (79, 80).

There are several published studies in which a relationship between insula function and interoception-related indexes, such as heart rate variability (HRV), has been reported. HRV refers to the fluctuations or changes in the interval between consecutive heartbeats and is considered to be a quantitative measure that can reveal irregular behavior of the heart and of dynamic, bi-directional heart–brain interactions (81, 82). An optimal level of HRV reflects healthy function and inherent self-regulatory capacity and adaptability (82, 83). Generally, HRV declines with age and decreased HRV is associated with various pathological conditions, especially cardiovascular disease (84) and psychiatric conditions (85). Over three decades ago it was proposed that the insula and other brain regions in the interoceptive network, such as anterior cingulate, hypothalamus, as well as prefrontal cortex, are key sites for modulating cardiac rhythm (86) and this is consistent with the findings of more recent resting-state fMRI studies in which increases in HRV, accompanied by higher functional connectivity between the insula and the prefrontal cortex (87) and amygdala (88), have been reported. In addition, HRV biofeedback induced increase in HRV was found to be associated with increased resting-state functional connectivity between insula and ventro-medial prefrontal cortex (89).

The finding that the interoceptive ability of an individual is closely related with insular activity and connectivity has been explored further in several clinical studies and especially of people with depression in whom interoceptive accuracy has been shown to be reduced (11) and HRV decreased (81, 90). For example, reduction in the volume and thickness of the insula (33, 34, 36), and hypo-connectivity of the insula within the affective networks and with the frontoparietal brain networks [see meta-analysis (91)], have been reported in people with MDD. In addition, hypo-activation of insula during various tasks requiring visceral interoceptive attention or recall were consistently observed in people with MDD (92–94). For example, compared to healthy controls people with depression were reported to show hypo-activation of anterior insula during heartbeat counting (92), and activity of bilateral dorsal mid-insular cortex was significantly reduced and negatively correlated with depression severity in unmedicated adults with MDD compared to healthy controls in an experiment where participants were instructed to focus on the intensity of sensations arising from the visceral organs including heart, stomach, and bladder (93). Moreover, when simply recalling a stimulus associated with a previous interoceptive perception, people with MDD also demonstrated reduced insular activity compared to healthy controls (94). Structural deficits, aberrant baseline functioning and abnormal interoceptive representation of the insula in people with depression may provide useful neuro-biomarkers for studying the disturbance of interoception and the reduced interoceptive ability in depression. Interestingly, when presented with a task to measure inhibitory control, people with depression showed a higher level of fronto-insular functional connectivity and which was associated with decreased HRV compared to healthy controls (90). In people with depression there appeared to be compensatory connections between interoceptive cortex and cortex responsible for higher-order cognitive function, which could point to a potential mechanism for cognitive control of interoceptive behavior in people with depression.

Greater interoceptive accuracy of cardiac perception has also been linked to increased levels of state and trait anxiety (80, 95) and HRV levels indicative of arrhythmias (82). Thus, an appropriate range of interoceptive perception ability and corresponding insular function is suggested to be crucial for both mental and physical health and well-being.

### 3.3. Interoceptive modulation

A variety of therapeutic methods and training that produce interoceptive modulation are available for treatment of people with depression and have been demonstrated to be effective in relieving symptoms and preventing relapse. Central to all the methods is that they advocate a shift from paying attention to the external environment to focusing on bodily sensation and strengthening connection between body and mind. Although the modulations in brain function that occur affect a diverse range of brain structures and networks, they have been consistently shown to modulate the insula, which is the primary hub for interoception (96).

Most of the therapies for interoceptive modulation are based on or have similarities to the philosophy of meditation, which emphasizes integration of body and mind, and because no universally accepted academic definitions have been made, meditation and mindfulness are often used in an undifferentiated way in referring to the treatments. Researchers have proposed several potential neurocognitive mechanisms for the therapeutic effect of interoceptive modulations on symptoms of depression and how these are related to the alterations in the insula.

Firstly, interoceptive modulation may relieve depression via restoration of impaired interoceptive functioning and which is likely to be mediated by changes in insular function. Interoceptive accuracy has been shown to be significantly decreased in depression and to be improved by brief or long-term interventions focusing on bodily signals (97–99), and support for these changes being mediated by the insula comes from observations of significantly enhanced activity within the insular region in both beginners and experts in meditation practice (100) and increased functional coherence within interoceptive regions, including insula, during actual meditation (101). The latest evidence showed that the insular function was modulated by 8 weeks interventions of mindfulness training, which was shown to be associated with increased Body Trusting, a subscale of MAIA questionnaire, in more severe depressive patients (102). Another particular form of interoceptive modulation which has been shown to alleviate symptoms of depression is massage therapy (103, 104). Eggart et al. (105) have proposed that the antidepressive effect of massage therapy is elicited by the affective properties of touch which stimulates C tactile afferents in the skin which project via the vagus nerve to the insular and other interoceptive regions. Perhaps, the most convincing evidence of the possible modulating effect of meditation on insula function comes from the findings that long-term mediators and TaiChi practitioners show greater grey matter thickness and density in the insula, especially in the anterior subregions, compared to controls (106–108). Thus, interoceptive modulation might produce neuroplasticity changes in insula leading to positive impacts on interoceptive ability and functioning, and further influence the associated emotional and mood states of individuals. In future, longitudinal studies of the neuroplasticity changes produced by meditation and their association with interoceptive functioning in people with depression are likely to be highly informative.

Secondly, interoceptive modulation is suggested to lead to improvements in self-referential thinking and with greater awareness. This suggestion is based on the observation that mindfulness-based cognitive therapy enhanced concentration ability, reflected in changes in event-related brain potentials, and shifted attention away from potentially depressive rumination in people with depression (109). Further support comes from a report of the uncoupling of the right insula and the medial prefrontal cortex (PFC), which is a core area in default mode network (DMN), and increased connectivity between the right insula and the dorsolateral PFC regions in individuals after mindfulness training (110). Altered connectivity, especially hyper-connectivity within the DMN, has been considered to be an important neuropathophysiological mechanism underlying maladaptive rumination in MDD (111). A reduction in insula-DMN connectivity and increased connectivity of the insula with dorsolateral PFC might produce positive changes in disordered self-referential thinking and more effective regulation of interoceptive functioning, leading to greater self awareness based on more objective analysis of interoceptive and exteroceptive experiences (100).

Thirdly, interoceptive modulations may alter neural responses to pain perception. For example, experienced yoga practitioners, who are readily able to tolerate thermal pain (cold or heat), have greater insular volumes compared to controls, and furthermore, insular volume was found to be positively correlated with level of thermal pain tolerance and duration of yoga experience, suggesting that yoga may produce neuroplastic change in the insula (112). Kakigi et al. (113) reported that a yoga master who claimed no pain at all during meditation, showed a remarkable difference between the meditative and non-meditative state in an fMRI study. In particular, there was little or no increase in brain activity in the key regions responsible for interoception (including the insula) following painful laser stimulation delivered during meditation whereas significant changes occurred in the non-meditative state. As reported above, people with depression show hyper-sensitivity to interoceptive pain and over activation of the insula during pain processing and it is plausible that interoceptive modulations might change the neural processing of pain by the insula and further reduce the accompanying negative emotional responses. Some caution is necessary however, in that Nakata et al. (114) reviewed previous studies which showed an increase or decrease in insular reactivity to pain stimuli, and reported that results may vary depending on the specific forms and duration of meditation practice. A further consideration is that experimentally induced pain is usually multi-dimensional and mediated by various neural pathways for perception and affect. Findings can rarely be interpreted from a single perspective. A better understanding of the components of different kinds of pain and their specific influences on human emotions and behavior is required in order to understand how changes of insula function may affect pain perception in treatment of depression.

Beyond the findings and proposed theories mentioned above, there are also some intriguing new areas in modern medicine relating to the study of interoceptive modulations in people with depression and which incorporate the insula as an important brain structure for further in-depth investigations.

One topic that has been brought into the spotlight is the interplay between the body and brain via the microbiota-gut-brain axis. In particular, it has been claimed that the vagus nerve system and the immune system are specific interoception-related pathways that have been implicated in the communication between the microbiota and the brain (115, 116), and preliminary studies in human infants and adults using resting-state fMRI have provided evidence for an association between insular connectivity and microbiome diversity, suggesting that the microbiota may play a role in shaping the brain during human development (117, 118). These results highlighted the potential role of the interoceptive pathway, with the insula involved, in the interplay between the microbiota and brain. Given the significant changes in the microbial population and diversity as well as a large anti-depressive effect of probiotics, which have been found in people with MDD (119, 120) it will be interesting in future to obtain more evidence of how the microbiota influences the development of interoceptive processing and the corresponding neuromechanism across the lifespan, and how probiotics in turn influence the emotion and change the symptoms of depression.

Neuromodulation, as a multidisciplinary term, is another topic that has been attracting increasing interests and becoming a significant area in neuroscience over the years. In psychiatric domains, neuromodulation has been employed as a specific form of treatment in MDD through the use of techniques, such as transcranial magnetic stimulation (TMS) and transcranial current electrical stimulation (tDCS), to directly stimulate the brain. More importantly, neuromodulation has emerged as a promising tool to achieve interoceptive modulation by manipulating brain activity in regions associated with interoceptive signal processing. Specifically, it has been proved that interoceptive processes could be modulated by stimulating the insula; for example, by using the technique of TMS with a modality of continuous theta-burst stimulation, the inhibition of the right anterior insula could result in a significant decline in both interoceptive accuracy and confidence judgements for cardiac and respiratory signals (121); for another example, disturbing the activity of insula by means of tDCS has been found to reduce the practice effect in interoceptive accuracy (122). Notably, stimulating conventional brain target used in repetitive TMS for MDD patients, such as left or right dorsolateral prefrontal cortex, could also influence remote insular connections which may be predictive of treatment response (123, 124). These results offer the possibilities for further study to target the insula as a brain stimulation area and to evaluate the optimal stimulating strategy, from the perspective of interoceptive modulation, for the treatment of MDD.

Taking all of the above findings into consideration, the insula is suggested to be a pivotal brain structure, and site of an underlying mechanism, through which interoception-related modulations can ameliorate depression.

## 4. Conclusion and future directions

The potential role of insular dysfunction in producing underlying interoceptive deficits in people with MDD has been reviewed. There is striking evidence, obtained from studies of the perception of heartbeats, of interoceptive dysfunction in people with depression. This interoceptive dysfunction has been linked to structural or functional impairments of the insula which plays a central role in processing of interoceptive information. Three aspects of the potential relationship between interoceptive dysfunction and alterations in insular function in people with depression have been assessed, namely clinical symptoms, quantitative measures of interoceptive function and ability, and interoceptive modulation. Firstly, increased severity of somatic symptoms in people with depression was found to be associated with impaired insular function, and among the somatic symptoms, fatigue and pain are the most prominent and have been considered in greatest detail. Secondly, the insula has been demonstrated to be a brain region the function of which is most closely related to the interoceptive ability of an individual. Insular hypo-activation has been reported in people with MDD who were asked to focus on attending to their visceral sensations, indicating a weaker involvement of the insula in processing interoceptive information. Thirdly, interoceptive modulation, produced by various treatments which emphasize attending to bodily sensations and the body–mind connection, produces neuroplastic changes in the insula, restores impaired interoceptive function, rebuilds self-referential thinking and reduces the neural response to pain, and relieves symptoms of depression.

These preliminary conclusions do however need to be tested in more detailed studies of people with MDD which will offer interesting insights into future work. Firstly, the particularly broad definition that is used for interoception should be acknowledged. For example, signals from the body and the external environment can both evoke changes in insula activity, thus blurring the line between interoception and exteroception (3). Accordingly, to gain a better understanding of interoceptive function, it is essential to conduct further research to create autonomic or objective measures that take into account activities of multiple internal organs, not just the cardiac-based ones, and to explore how disturbed interoceptive function may link to insular changes and how it could be a factor in the clustering of symptoms of depression. Secondly, research has emerged to explore the elements that have an effect on individual interoceptive ability. A recent study revealed that early life traumatic experiences may alter individual emotion regulation capacities by influencing their interoceptive functions (e.g., interoceptive sensibility) (125). Therefore, further research is necessary and beneficial for the study of MDD to explore the shaping and development of individual interoception throughout the lifespan and its influencing factors, as well as to delve deeper into the dynamic role of the brain insula in it. In addition, although the relationship between large-scale brain connectivity and interoceptive dysfunction in people with depression has been studied, there is still little information regarding underlying cellular and synaptic processes. Lastly, we propose a ‘dual-track’ interoceptive modulation strategy for the treatment of MDD, which consists of both peripheral and central regulation. Peripheral regulation focuses on triggering an effect on afferent interoceptive signaling, e.g., through gut microbiota-derived mediators. Central regulation, on the other hand, seeks to directly stimulate the brain. We hypothesize that both approaches point to insular modulation, which underlies the neuromechanism of interoceptive enhancement in the treatment of MDD.

In conclusion, evidence from the existing studies has shown that the insula may be the central structure for the impaired interoceptive function as identified in people with depressive disorder. Future systematic assessments of interoceptive dysfunction and their association with insular function in those with depressive disorder are likely to be highly important in the treatment of MDD.

## Author contributions

LH: Conceptualization, Writing – original draft, Writing – review & editing, Validation, Funding acquisition, Investigation. HH: Conceptualization, Validation, Writing – review & editing, Writing – original draft. CL: Writing – review & editing. NR: Writing – review & editing. JC: Writing – review & editing. GY: Writing – review & editing. LP: Writing – review & editing. XS: Writing – review & editing.

## Funding

The author(s) declare financial support was received for the research, authorship, and/or publication of this article. This work was supported by STI 2030-Major Projects (No. 2022ZD0208500); National Natural Science Foundation of China (Nos. 82101620, 62006038 and U2033217); Chengdu Municipal Health Commission (No. 2021365); and Medical Science and Technology Project of Sichuan Provincial Health Commission (21PJ148).

## Conflict of interest

The authors declare that the research was conducted in the absence of any commercial or financial relationships that could be construed as a potential conflict of interest.

## Publisher’s note

All claims expressed in this article are solely those of the authors and do not necessarily represent those of their affiliated organizations, or those of the publisher, the editors and the reviewers. Any product that may be evaluated in this article, or claim that may be made by its manufacturer, is not guaranteed or endorsed by the publisher.
